# Subcellular Localization of Galloylated Catechins in Tea Plants [*Camellia sinensis* (L.) O. Kuntze] Assessed via Immunohistochemistry

**DOI:** 10.3389/fpls.2016.00728

**Published:** 2016-05-26

**Authors:** Huanhuan Xu, Ya Wang, Yana Chen, Pan Zhang, Yi Zhao, Yewei Huang, Xuanjun Wang, Jun Sheng

**Affiliations:** ^1^Key Laboratory of Pu-er Tea Science, Ministry of Education, Yunnan Agricultural UniversityKunming, China; ^2^Tea Research Center of YunnanKunming, China; ^3^College of Food Science and Technology, Yunnan Agricultural UniversityKunming, China; ^4^College of Pu-er Tea, Yunnan Agricultural UniversityKunming, China; ^5^State Key Laboratory for Conservation and Utilization of Bio-Resources in YunnanKunming, China

**Keywords:** *Camellia sinensis*, catechins, galloylated catechins, subcellular localization, immunohistochemistry, HPLC

## Abstract

Galloylated catechins, as the main secondary metabolites in the tea plant, including (-)-epigallocatechin-3-gallate and (-)-epicatechin-3-gallate, comprise approximately three-quarters of all the tea plant catechins and have stronger effects than non-galloylated catechins, both on the product quality in tea processing and the pharmacological efficacy to human beings. The subcellular localization of galloylated catechins has been the primary focus of studies that assess biosynthesis and physiological functions. Classical histochemical localization staining reagents can not specifically detect galloylated catechins; thus, their subcellular localization remains controversial. In the present study, we generated a monoclonal antibody (mAb) against galloylated catechins, which can be used for the subcellular localization of galloylated catechins in the tea plant by immunohistochemistry. Direct ELISA and ForteBio Octet Red 96 System assay indicated the mAb could recognize the galloylated catechins with high specificities and affinities. In addition, tea bud was ascertained as the optimal tissue for freezing microtomic sections for immunohistochemistry. What’s more, the high quality mAbs which exhibited excellent binding capability to galloylated catechins were utilized for the visualization of them via immunohistochemistry. Our findings demonstrated that vacuoles were the primary sites of localization of galloylated catechins at the subcellular level.

## Introduction

Tea is one of the most consumed beverages globally for centuries (Huang et al., 2014), second only to water. It is produced from tea plants [*Camellia sinensis* (L.) O. Kuntze] buds (Kim et al., 2013b; Schramm, 2013; Sonoda et al., 2014). Tea infusion contains abundant nutrients and functional ingredients, which are beneficial to the human health (El-Shahawi et al., 2012); specifically, the tea plant polyphenols have many important physiological functions (Liu et al., 2012). Catechins, consisting the most important flavonoid group, account for approximately 80% of all the polyphenols, which in turn constitute approximately 18–36% of the dry leaf weight. The main catechins are (-)-epicatechin (EC), (-)-epigallocatechin (EGC), (-)-epicatechin-3-gallate (ECG), and (-)-epigallocatechin-3-gallate (EGCG; Liu et al., 2009; Roman, 2013; Lu et al., 2014). It is notable that galloylated catechins account for approximately 76% of all catechins, of which EGCG is the most abundant, active, and thoroughly investigated, followed by ECG. Their mechanism of action indicates that the polyphenolic structure of galloylated catechins makes them good donors for hydrogen bonding, which increases the water solubility of galloylated catechins and contributes to their high affinity to proteins and nucleic acids (Yang et al., 2007).

Galloylated catechins have stronger health effects as compared to the non-galloylated catechins, with respect to the product quality in tea processing and pharmacological efficacy in humans (Bigelow and Cardelli, 2006; Tsutsumi et al., 2011). Galloylated catechins possess antioxidant, antibacterial, and anti-inflammatory effects and alleviate cardiovascular diseases (Braicu et al., 2011; Jin et al., 2013; Kim et al., 2013a; Moravcova et al., 2014); however, their subcellular localization have not been fully addressed (Suzuki et al., 2003; Liu et al., 2009, 2012). Previous studies have demonstrated that catechins were mainly located in the chloroplasts or vacuoles at the subcellular level (Suzuki et al., 2003; Liu et al., 2009). These finding were largely attributable to the methodologies applied, each having a unique set of advantages and limitations. Although, the classical histochemical localization staining reagents, including vanillin-HCl, diazotized amines, 4-dimethylaminocinnamaldehyde (DMACA), and potassium permanganate (Feucht et al., 1992; Liu et al., 2009; Bhattacharya et al., 2015), are extensively used to determine the subcellular localization of polyphenols, they do not specifically detect the galloylated catechins. It is well known that immunohistochemistry could be used to localize small molecules specifically. Moreover, this method has been successfully used to localize some secondary metabolites in plant. Immunohistochemical localization with primary anti-caffeine antibodies and conjugated secondary antibodies on tea leaf sections proved at the tissue level that caffeine was localized and accumulated within vascular bundles, mainly the precursor phloem (van Breda et al., 2013). Similarly, delta 9-tetrahydrocannabinol (THC) localization in glandular trichomes and bracteal tissues of *Cannabis* was examined with a mAb, which can specifically recognize THC (Kim and Mahlberg, 1997). Therefore, the subcellular localization of galloylated catechins in the tea plant by mAb as a rapid and sensitive method is necessary.

Precise information regarding the subcellular localization of galloylated catechins in the tea plant would be helpful for understanding how their biosynthesis is controlled, thereby providing additional insights into the related metabolic pathways. Advances in the high specificity of immunohistochemistry have prompted us to probe the subcellular localization of galloylated catechins in the tea plant. In this content, the anti-galloylated catechin mAb was generated. The high galloylated catechin content tissues were subsequently assessed via high-performance liquid chromatography (HPLC), and the determination of the subcellular localization of galloylated catechins was determined using immunohistochemistry.

This study reports production of a anti-galloylated catechin mAb which can recognize the galloylated catechins with high specificities and affinities. The high quality mAbs which exhibited excellent binding capability to galloylated catechins were further used to localize them in the tea plant. Our findings demonstrated that vacuoles were the primary sites of localization of galloylated catechins at the subcellular level, which is intuitive for their underlying mechanisms of biosynthesis, transport, storage, and physiological functions. Moreover, the subcellular localization of galloylated catechins in the tea plant assessed via immunohistochemistry may contribute toward a theoretical reference for the assessment of the subcellular localization of secondary metabolites in plants.

## Materials and Methods

### Chemicals and Reagents

Epigallocatechin-3-gallate, ECG, EGC, EC, gallic acid (GA), and resveratrol (RSV) were of high purity grade (≥98%) and purchased from Aladdin (Shanghai, China). Culture media and serum were obtained from Thermo Fisher Scientific (Waltham, MA, USA) and Biological Industries Israel Beit Haemek Ltd., respectively. Optimal Cutting Temperature (OCT) Compound was purchased from Sakura Finetek, USA. Formalin was obtained from Jinan Biological Technology Co., Ltd. Paraformaldehyde (PFA) was obtained from the Tianjin Branch of Chemical Reagent Co., Ltd (Tianjin, China). The ABC high-HRP immunostaining kit was manufactured by Vectastain Co., Ltd. The haematoxylin stain was of high purity grade and was purchased from Sigma-Aldrich (St Louis, MO, USA). Normal mouse IgG (sc-2025) was obtained from Santa Cruz Biotechnologies (Santa Cruz, CA, USA). The HRP-DAB Chromogenic Substrate Kit was obtained from Tiangen Biotech (Beijing, China) Co., Ltd. Neutral red was obtained from Shanghai Yuanye Biological Technology Co., Ltd (Shanghai, China). Acetonitrile and trifluoroacetic acid used in the mobile phases were of HPLC grade and procured from Tedia Co., Inc. (Fairfield, OH, USA) and Merck KGaA (Darmstadt, Germany), respectively.

### Plant Materials

Tea plant [*C. sinensis* (L.) O. Kuntze] samples were obtained from the Yunnan Agricultural University, Kunming, China. Each sample included bud, first leaf, second leaf, third leaf, and tender stem, which were separated from the tea plant. The samples were maintained at 4°C to inhibit air contact prior to analysis.

### Animals

Healthy male BALB/c mice (6–8 weeks old) were purchased from Nanjing Peng-sheng Biotechnology Co., Ltd, China. All mice were housed in polypropylene cages with sterile paddy husk; the animals were maintained under ambient conditions of temperature and humidity with a 12 h light/dark cycle. Food and water were provided *ad libitum*. All mice experiments were performed in the animal facility according to the institutional guidelines and were approved by the Institutional Animal Care and Use Committee of Yunnan Agricultural University. The physical condition of the animals was monitored daily, and any adverse events were not observed. The animals were anesthetized prior to immunization, and spleen cells were obtained from the immunized mice, which were euthanized via cervical dislocation following CO_2_ anesthesia.

### Production of Monoclonal Antibodies

Two BALB/c mice were immunized with oxidized tea polyphenols (OTP), which contains EGCG as the major group (Huang et al., 2014). Briefly, each mouse was vaccinated five times with 40 μg of OTP at a 2-week interval. The first immunization was performed using Freund’s complete adjuvant; the incomplete adjuvant was used for the subsequent immunizations. Following the immunization procedure, spleen cells from the immunized mice were mixed with myeloma SP2/0 cells at a ratio of 5:1. The mixture was washed twice with pre-warmed RPMI-1640 (37°C) and pre-warmed 50% polyethylene glycol (PEG) 1500 (Sigma–Aldrich, Germany) was used for fusion. A HAT selective medium (Sigma–Aldrich, Germany) was consecutively used for the selection of hybridoma cells. Direct Enzyme-linked immunosorbent assay (ELISA) was performed to screen the EGCG positive hybridoma cells, with the plates coated with EGCG during the cloning of EGCG hybridomas. One hybridoma clone, P6C2, was selected from 326 clones raised against EGCG. Positive hybridomas were cultured for 2 weeks. The anti-galloylated catechin antibody was purified from the culture supernatants via affinity chromatography using a Pierce^TM^ protein A/G column (Bayat et al., 2013). The eluted antibody was dialyzed against phosphate buffered saline (PBS) at pH = 7.2. SDS-PAGE analysis was performed following the Laemmli method, with a 12.0% separating gel to assess the purity and molecular weight of the mAb. The gel was stained with 0.25% Coomassie Brilliant Blue G-250.

### Amino Acid Sequence of the Light Chain Variable Region Gene in the P6C2 Monoclonal Antibody

RNA was extracted from P6C2 hybridoma cells by RNeasy (Qiagen, Inc.). Reverse transcription was performed using Superscript^TM^ III (Invitrogen, Inc.). cDNA fragments of the P6C2 light chain variable region gene were amplified via PCR using Fast-Pfu (Transgene, Beijing, China) with universal primers (Wang et al., 2000) as follows:

F-Kc: 5′-ACAAATGCCTATGCAGAYATTGTGMTSACMCARWCTMCA-3′, R-Kc: 5′-GGATACAGTTGGTGCAGCATC-3′.

The amplified PCR fragment was cloned into the pBR322 vector with an infusion kit (Clontech Inc., Mountain View, CA, USA) according to the manufacturer’s protocol. The DNA insert was sequenced by Invitrogen (Shanghai, China), and the primary structure of the kappa chain variable region was deduced.

### Antibody Specificity Analysis

**FIGURE 1:**
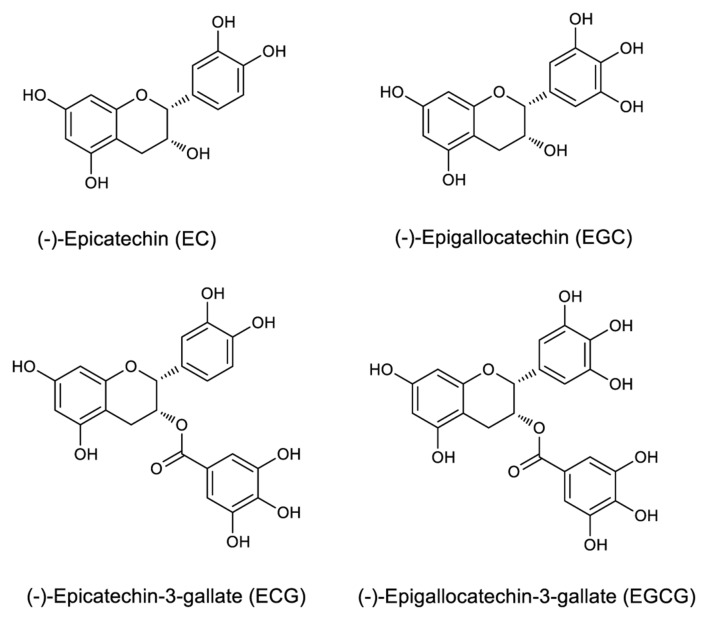
**Structures of selected catechins**.

Epicatechin, EGC, ECG, and EGCG have a basic 2-phenylchromone structure (**Figure 1**), and non-galloylated catechins (EC and EGC) and GA are the precursor substances of galloylated catechins (ECG and EGCG). These compounds and RSV were used to evaluate the mAb’s specificity. Direct ELISA was performed on 96-well immunoplate (Nunc Polysorp; Thermo Fisher Scientific), which were coated with EGCG, ECG, EGC, EC, GA, and RSV (at the same concentration, 50 μg mL^-1^) overnight at 4°C and then blocked by incubation with 1% bovine serum albumin (BSA). The mAbs were applied to duplicate wells, followed by HRP-conjugated goat anti-mouse IgG (Thermo Fisher Scientific, Waltham, MA, USA) as the secondary antibody. Tetramethylbenzidine (TMB) high sensitivity substrate solution (BioLegend, San Diego, CA, USA) was added subsequently and the absorbance was recorded at 450 nm using an ELISA reader (Thermo Fisher Scientific, Waltham, MA, USA).

Furthermore, the interactions between the anti-galloylated catechin antibody and EGCG, ECG, EGC, EC, and GA, were determined using Super Streptavidin (SSA) biosensors in an Octet Red 96 instrument (ForteBio Inc., Menlo Park, CA, USA). Interactions were assessed at 30°C in PBS (pH = 6.5) unless otherwise stated. Briefly, the antibody was biotinylated at a 1:1 stoichiometry for 1-h at room temperature (RT). The biotinylated antibody was subsequently desalinated by a PD SpinTrap^TM^ G-25 column (GE Healthcare, UK). SSA tips were pre-wet for 10 min in buffer immediately prior to use, and the 96-well black plates utilized in the Octet were filled with 200 μL of sample or buffer per well and agitated at 1000 rpm. The antibody was loaded to the SSA biosensors, which were washed with the buffer for 120 s and transferred to the wells that contained the compounds.

### Antibody Binding Affinity Determined by Octet

The binding affinity of anti-galloylated catechin antibody with EGCG or ECG was determined using SSA biosensors in the Octet Red 96 instrument. The biotinylated antibody and EGCG or ECG at different concentrations were prepared in the assay buffer (PBS, pH = 6.5) as described above. We measured EGCG and ECG association and dissociation for 10 min, respectively. The kinetic parameters and affinities were calculated from a non-linear global fit of the data between the galloylated catechins and the antibody, using Octet Data Analysis software version 7.0 (ForteBio Inc., Menlo Park, CA, USA).

### HPLC Analysis

The required sample amounts were crushed under liquid N_2_ to increase the extraction yield. An 80% methanol–water mixture was used as the extraction solvent (El-Shahawi et al., 2012; Otles and Yalcin, 2012). HPLC analysis was performed using a linear gradient system with mobile phase A (100% acetonitrile) and mobile phase B (0.03% trifluoroacetic acid), which were filtered through a 0.45 μm filter. The mobile phase A was increased from 10 to 60% in 36 min (Ning et al., 2009). A 10 μL sample was analyzed using an auto sampler (G1329B, 1260ALS, Agilent, USA), an ultraviolet detector (G1314F, 1260VWD, Agilent, USA) at 280 nm, and a HPLC pump (G1311B, 1260Quat Pump, Agilent, USA) at 1.0 mL min^-1^ flow at 40°C (G1316A, 1260TCC, Agilent, USA) through a C_18_ ODS column (ZORBAX SB-C_18_ 4.6 mm × 250 mm, 5 microns, Agilent, USA).

### Tissue Slicing and Immunohistochemistry

Fixation, embedding, sectioning, and staining of tea tissues were performed using the standard methods. Briefly, the tea tissues were fixed for 24 h at RT in 10% neutral buffered formalin. The OCT embedded tissues were cut into 20 μm sections using a Cryostat Microtome (Leica, Germany). The slides were fixed with 4% PFA for 10 min at 4°C and blocked with Blocking Solution (SP-6000, Vector Laboratories, Inc.) for 20 min at 37°C. After blocking for 30 min at 37°C with normal serum, histological sections of the tea tissues were incubated overnight at 4°C with the anti-galloylated catechin antibody. The sections were then sequentially incubated with biotinylated antibody and the ABC reagent for 30 min at 37°C. The reaction was visualized with DAB, and counterstaining was conducted with haematoxylin for 5 min. The sections were subsequently analyzed via fluorescence microscopy (Leica). Images of the tea tissue sections were acquired before and after staining.

### Statistical Analyses

All values were presented as mean ± standard errors of the mean (SEM). All analyses were performed using SPSS 17.0 (Chicago, IL, USA), Graphpad Prism 5 (Graphpad Software, Inc., La Jolla, CA, USA) and Octet Data Analysis software version 7.0 (ForteBio Inc., Menlo Park, CA, USA).

## Results

### Antibody Expression

**FIGURE 2:**
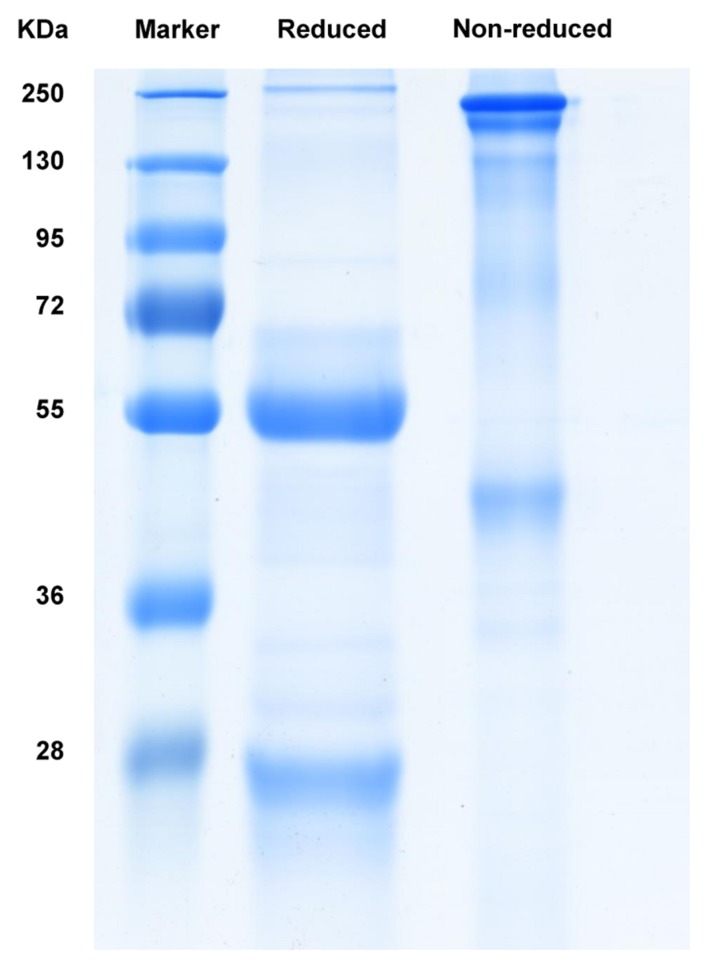
**SDS-PAGE analysis of the purified mAb following Coomassie blue staining**.

The anti-galloylated catechin antibody was expressed in hybridoma cells and purified by affinity chromatography using a Pierce^TM^ protein A/G column. The SDS-PAGE analysis of the purified mAb demonstrated the whole protein in the non-reducing conditions (~150 kDa) and 50 and 25 kDa heavy and light chains, respectively, in the reducing conditions (**Figure 2**).

### Amino Acid Sequence of the Kappa Chain Variable Region of the mAb

The sequence of the P6C2 kappa chain variable region was deduced from the sequence of DNA cloned into the pBR322 vector, as shown in Supplementary Figure S1. According to NCBI-blast, it was closely associated with the kappa chain variable region of the immunoglobulin raised under chloramphenicol selection.

### Specificity of Antibody

**FIGURE 3:**
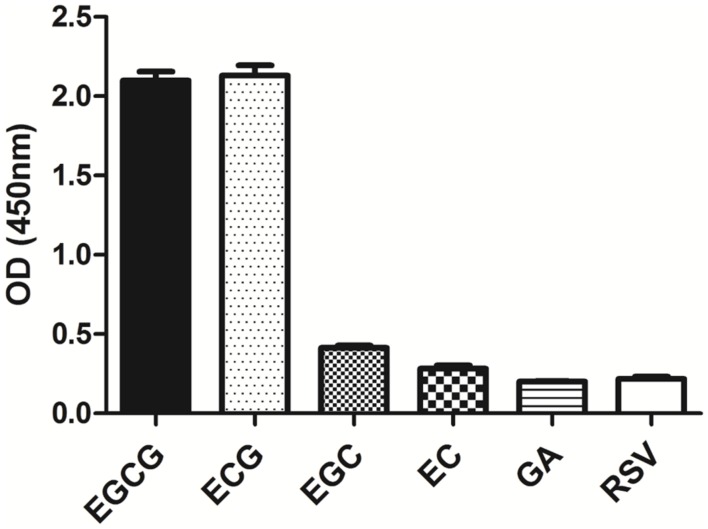
**Determination of specificity of the anti-galloylated catechin mAb by direct ELISA.** The anti-galloylated catechin mAb was tested against epigallocatechin-3-gallate (EGCG), epicatechin-3-gallate (ECG), epigallocatechin (EGC), epicatechin (EC), GA (gallic acid), and resveratrol (RSV) at the same concentration (50 μg mL^-1^). Data were presented as mean ± SEM of three independent experiments.

**FIGURE 4:**
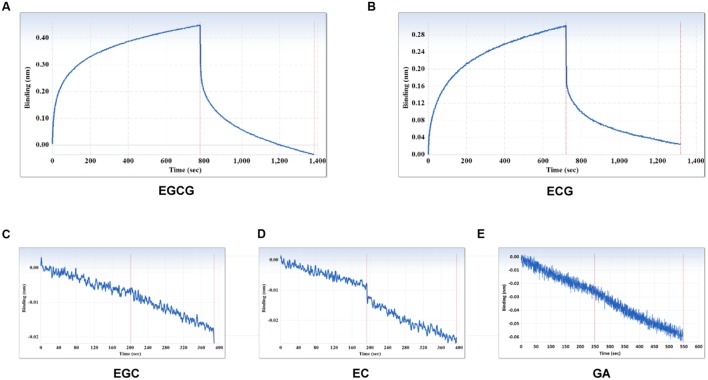
**Interactions between the main compounds and anti-galloylated catechin antibody assessed via the label-free ForteBio Octet Red 96 System assay. (A)** EGCG; **(B)** ECG; **(C)** EGC; **(D)** EC; **(E)** GA. Concentration, 50 μM. Curves correspond to the phases of association and dissociation.

The specificity of the purified mAb was assessed by direct ELISA on EGCG, ECG, EGC, EC, GA (tea polyphenols), and RSV (non-tea polyphenol; **Figure 3**). Our finding showed that the mAb specifically recognized EGCG and ECG, rather than EGC, EC, GA, and RSV. Besides, the specificity of the mAb was identified by Bio-Layer Interferometry (BLI) technique. The real-time interactions between the mAb and precursor substances of galloylated catechins, including the main non-galloylated catechins (EC and EGC) and GA, were determined using the ForteBio Octet system (ForteBio Inc., Menlo Park, CA, USA; **Figure 4**). The curves correspond to the phases of association and dissociation. In the association steps, as anticipated, EGCG and ECG could significantly bind to the anti-galloylated catechin antibody (**Figures 4A,B**), the binding signal values were gradually increased and finally achieved 0.40 and 0.28 nm, respectively. Whereas there were scarcely any signals in the interactions between the mAb and EGC, EC, and GA (**Figures 4C–E**), which means that the mAb could not recognize them. In consideration of catechin structures, EGCG and ECG contain a GA moiety at position 3 on the C ring, whereas other compounds (EGC, EC, and GA) do not contain this functional group (**Figure 1**), that may be the reason why the binding curves for EGCG and ECG are so different from the binding curves for EGC, EC, and GA. It indicates that the GA moiety functional group is necessary for anti-galloylated catechin antibody binding. The results above showed that the mAbs were capable of recognizing galloylated catechins with high specificity.

### Evaluation of Binding Affinity of mAbs

**FIGURE 5:**
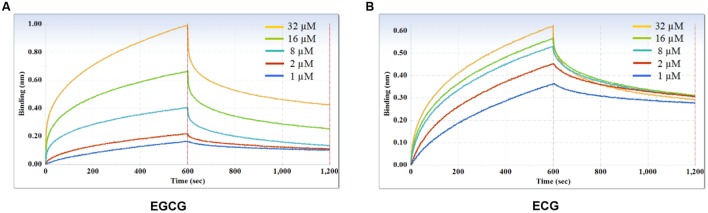
**Binding affinity measurements of anti-galloylated catechin antibody with galloylated catechins assessed via the ForteBio system. (A)** EGCG; **(B)** ECG. Curves correspond to the phases of association and dissociation of galloylated catechins at various concentrations on the monoclonal antibody anchored to the sensor chip. These curves may be used to determine the *K*_D_, *K*_on_, and *K*_off_.

Furthermore, kinetic studies were performed to determine the binding affinities of the anti-galloylated catechin mAbs to serially diluted galloylated catechins from 32 to 1 μM (**Figure 5**; **Table 1**). The on-rate kinetic (*K*_on_) values were displayed by EGCG at 2.27*E* + 02 Ms^-1^ and ECG at 2.65*E* + 03 Ms^-1^, respectively; and the off-rate kinetic (*K*_off_) values were displayed by EGCG at 2.10*E* – 03 s^-1^ and ECG at 5.14*E* – 04 s^-1^, respectively. To understand the binding affinity value, the equilibrium dissociation constant (*K*_D_) was calculated by *K*_off_/*K*_on_. This showed that the mAb had strong affinity to EGCG and ECG, with the *K*_D_ values of 9.25*E* – 06 and 1.97*E* – 07 M, respectively. The results indicated that the galloylated catechins had strong binding abilities with the anti-galloylated catechin antibody.

**Table 1:** Binding kinetics of the anti-galloylated catechin antibody to EGCG and ECG using the ForteBio system.

Variant	*K*_D_ (M)	*K*_on_ (1/Ms)	*K*_off_ (1/s)	*R*^2^
EGCG	9.25 - 06	2.27E + 02	2.10E - 03	0.893
ECG	1.97E - 07	2.65E + 03	5.14E - 04	0.694

*Off-rate kinetics (*K*_off_) were measured by saturating the SSA with the anti-galloylated catechin antibody and monitoring the dissociation after switching to buffer. The on-rate kinetics (*K*_on_) were measured using different concentrations of EGCG or ECG. *K*_D_, the equilibrium dissociation constant, was calculated as *K*_off_/*K*_on_, EGCG, ECG (**Figure 5**).*

### Identification of High Galloylated Catechin Content Tissues

To identify the optimal tissue for the assessment of the subcellular localization of galloylated catechins via immunohistochemistry, high galloylated catechin content tissues were ascertained using HPLC. Peaks were identified by retention time compared with EGCG and ECG standards, and calibration curves were performed using serially diluted standards (Supplementary Figure S2). The total galloylated catechin amounts were highest in the first tea leaf, followed by the tea bud, second leaf, and third leaf, with only small quantities of galloylated catechins in the tender stem (Supplementary Figure S3; **Table 2**). The EGCG content was higher as compared to the ECG, which is consistent with previous studies. Furthermore, the galloylated catechins were mainly distributed in the tea plant’s tender shoots.

**Table 2:** Galloylated catechin content (percentage of dry weight) of different tea tissues^a^.

Tissue	EGCG	ECG	SUM
First leaf	14.17 ± 0.15	8.80 ± 0.16	22.97 ± 0.31
Bud	11.78 ± 0.17	8.86 ± 0.19	20.64 ± 0.36
Second leaf	7.38 ± 0.15	3.62 ± 0.02	11.00 ± 0.17
Third leaf	2.88 ± 0.05	1.68 ± 0.02	4.56 ± 0.07
Tender stem	0.72 ± 0.00	0.45 ± 0.01	1.17 ± 0.01

*^a^Data were presented as mean ± SEM of three independent experiments.*

### Subcellular Localization of Galloylated Catechins via Immunohistochemistry

**FIGURE 6:**
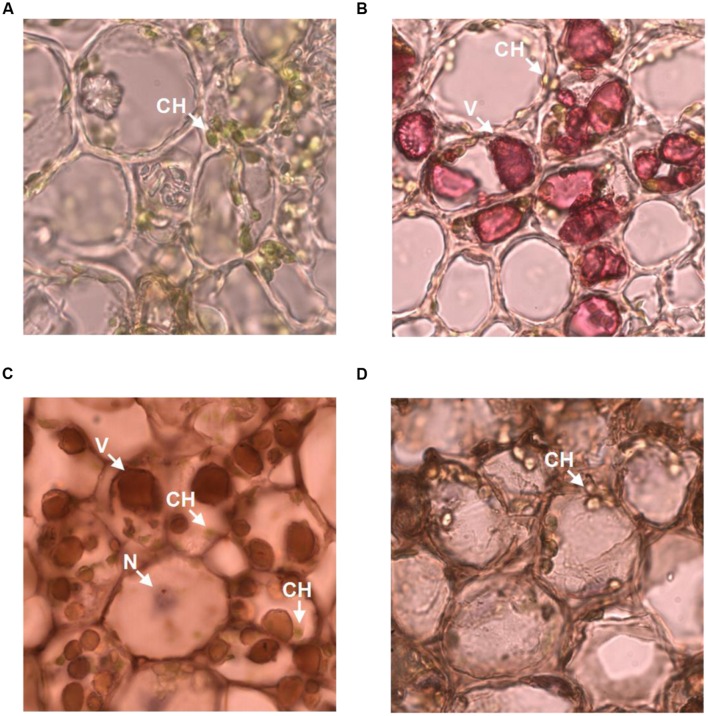
**Subcellular localization of galloylated catechins in the tea bud. (A,B)** Transverse bud sections before and after 0.5% neutral red staining, with red indicating the vacuoles. **(C,D)** Transverse bud sections after anti-galloylated catechin antibody and normal IgG antibody staining, respectively. Sepia represents galloylated catechin-accumulation areas. CH, chloroplast; V, vacuole; N, nucleus. Slice thickness, 20 μm. Oil microscopy.

The observation that anti-galloylated catechin antibody specifically recognized galloylated catechins prompted us to probe the subcellular localization of these compounds in the tea plant. The tea bud is a galloylated catechin-rich tissue and is suitable for frozen section preparation. Thus, it was selected for neutral red staining and immunohistochemistry. Using both the methods, sharp microphotographs were acquired (**Figure 6**). Vacuole localization was typically examined by neutral red staining since it specifically reacted with the plant vacuoles to yield a red complex. Following neutral red staining, the red-stained vacuoles in the tea bud were easily detected at the subcellular level, and the staining was not observed in the plastids (**Figures 6A,B**). The anti-galloylated catechin antibody was successively used to detect the subcellular localization of the galloylated catechins via immunohistochemistry. Interestingly, sepia-stained galloylated catechins in the tea bud were easily detected in vacuoles, whereas the chloroplasts were not stained (**Figure 6C**). This finding indicated that the galloylated catechins accumulated mainly in the vacuoles.

To validate the immunohistochemistry data, a normal IgG antibody was selected as a control. Any general or plastid staining was not identified (**Figure 6D**). Similarly, no staining was identified in plastids. Taken together, these findings suggested that vacuoles are the main sites for galloylated catechins at the subcellular level. Finally, these data supported immunohistochemistry as a novel, specific, and sensitive method for the assessment of the subcellular localization of galloylated catechins in tea plant samples.

## Discussion

Immunohistochemistry is widely used in biomedical research to localize specific epitopes of molecules in cells and tissues (Hewitt et al., 2014). In addition, immunohistochemistry is advantageous for the localization of small molecules because the technique is substantially more specific than other standard histochemical methods using stains, such as vanillin-hydrochloric acid, which are not unique for a distinct small molecule, but rather an entire class of compounds, and false positives or side reactions may occur. Furthermore, immunohistochemistry has been used to determine effectively the localization of caffeine in young tea leaves (van Breda et al., 2013). Monoclonal antibodies are a superior choice for immunohistochemistry because they detect only one epitope in a particular antigen. Small changes in the compound of interest structure as a result of fixation protocols typically do not affect the binding affinity. In the present study, OTP, which contains EGCG as the major group, was first used for production of the anti-galloylated catechin mAb. The specificities of the mAbs were checked by direct ELISA and BLI assays, and, the binding affinities of the mAbs with EGCG and ECG were determined using ForteBio Octet Red 96 System. Most importantly, the anti-galloylated catechin mAb was used for the subcellular localization of galloylated catechins in the tea plant. Our experimental results indicated that the specific binding of the mAb to galloylated catechins with high affinities could be used to investigate efficiently the subcellular localization of these compounds via immunohistochemistry. Considering catechin structures, what’s more, our findings suggest that the R1 galloyl group is necessary for anti-galloylated catechin antibody binding because both EGCG and ECG are the only catechins that contain this functional group (**Figure 1**). Therefore, immunohistochemistry is a useful method for assessing the subcellular localization of galloylated catechins in the tea plant. Using immunohistochemistry for galloylated catechins and neutral red staining for vacuoles, we demonstrated that the vacuoles were the primary sites for galloylated catechins at the subcellular level.

Although the galloylated catechins are the key secondary metabolites in the tea plant, the subcellular localization of galloylated catechins remains controversial. Using vanillin-HCl staining, Liu et al. (2009) demonstrated that catechins were primarily localized in the chloroplasts of mesophyll cells. However, catechins were simultaneously demonstrated to concentrate in callus vacuoles. In addition, Suzuki et al. (2003) demonstrated that catechins were primarily stored in the central vacuoles of the mesophyll cells as determined by Fe (II) staining. Till date, the subcellular localization of galloylated catechins remains unsolved, that is because none of the staining reagents could specifically detect galloylated catechins. Plant cells are distinguished from other eukaryotic cells by a rigid cellulose-based cell wall, chloroplasts, and a large central vacuole (Chi et al., 2015). The secondary metabolites may be considered chemical defense compounds against herbivores or microorganisms and are thus important for plant fitness. Several secondary metabolites are also toxic to the plant; thus, they need to be stored in a separate compartment (Wink, 1993). Polyphenol oxidase (PPO) is a ubiquitous copper metalloprotein in many plants, localized in chloroplasts, whereas flavonoid biosynthesis is speculated to occur in the cytoplasm (Roberts and Wood, 1950; Bokuchava et al., 1970; Buzun et al., 1970; Ono et al., 2006). We deduced that chloroplasts might not be the prime sites for galloylated catechin accumulation in the case of oxidization. As previously discussed, vacuoles were the primary sites for galloylated catechin accumulation at the subcellular level.

Recent studies have focused on the subcellular localization of galloylated catechins in the tea plant because these compounds possessed diverse biological activities and functions. Furthermore, multiple studies have identified promising preventative and therapeutic roles for galloylated catechins (Betts and Wareham, 2014; Chen et al., 2014; Cocci et al., 2014; Hodgson et al., 2014; Kim et al., 2014). Thus, the precise subcellular localization of galloylated catechins appears to be critical for unlocking valuable information regarding their biological roles. Our findings demonstrated that galloylated catechins accumulated in tea bud vacuoles, which indicates that vacuoles have a robust capacity to accumulate galloylated catechins. To assess the mode of transportation of synthesized galloylated catechins, sieve tube fluid of new shoots of tea plants was prepared via chelator-facilitated exudation (Dinant and Kehr, 2013), and the galloylated catechin content was determined by HPLC. Interestingly, galloylated catechins were detected in the sieve tube fluid (approximately 0.05% of the total amounts), which indicated that galloylated catechins may be transported through the sieve tube to vacuoles. The relative transportation mechanism need to be further studied.

## Conclusion

This study reports production of a anti-galloylated catechin mAb which can recognize the galloylated catechins with high specificities and affinities. The high quality mAbs which exhibited excellent binding capability to galloylated catechins were further used to localize them in the tea plant. Our findings demonstrated that vacuoles were the primary sites of localization of galloylated catechins at the subcellular level.

## Author Contributions

JS, XW, and YH conceived and designed the experiments. HX, YW, YC, PZ, YZ, and YH performed the experiments. HX, YH, and XW analyzed the data. JS and XW contributed reagents/materials/analysis tools. HX, XW, and YH wrote the manuscript. All authors read and approved the final manuscript.

## Conflict of Interest Statement

The authors declare that the research was conducted in the absence of any commercial or financial relationships that could be construed as a potential conflict of interest. The reviewer JPV and handling Editor declared their shared affiliation and the handling Editor states that the process nevertheless met the standards of a fair and objective review.
