# Supplementary figures and images for: Subcellular Localization of Galloylated Catechins in Tea Plants [Camellia sinensis (L.) O. Kuntze] Assessed via Immunohistochemistry
Source: Front Plant Sci. 2016 May 26;7:728. doi: 10.3389/fpls.2016.00728 (PMC4881381; doi:10.3389/fpls.2016.00728)

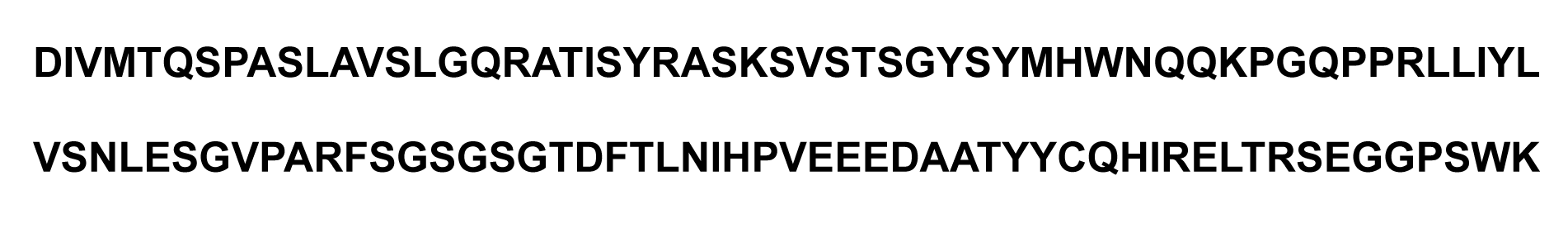

Supplement: Supplementary file 1 [file Presentation_1.ZIP › Supplementary Material/Supplementary figure 1.tif]

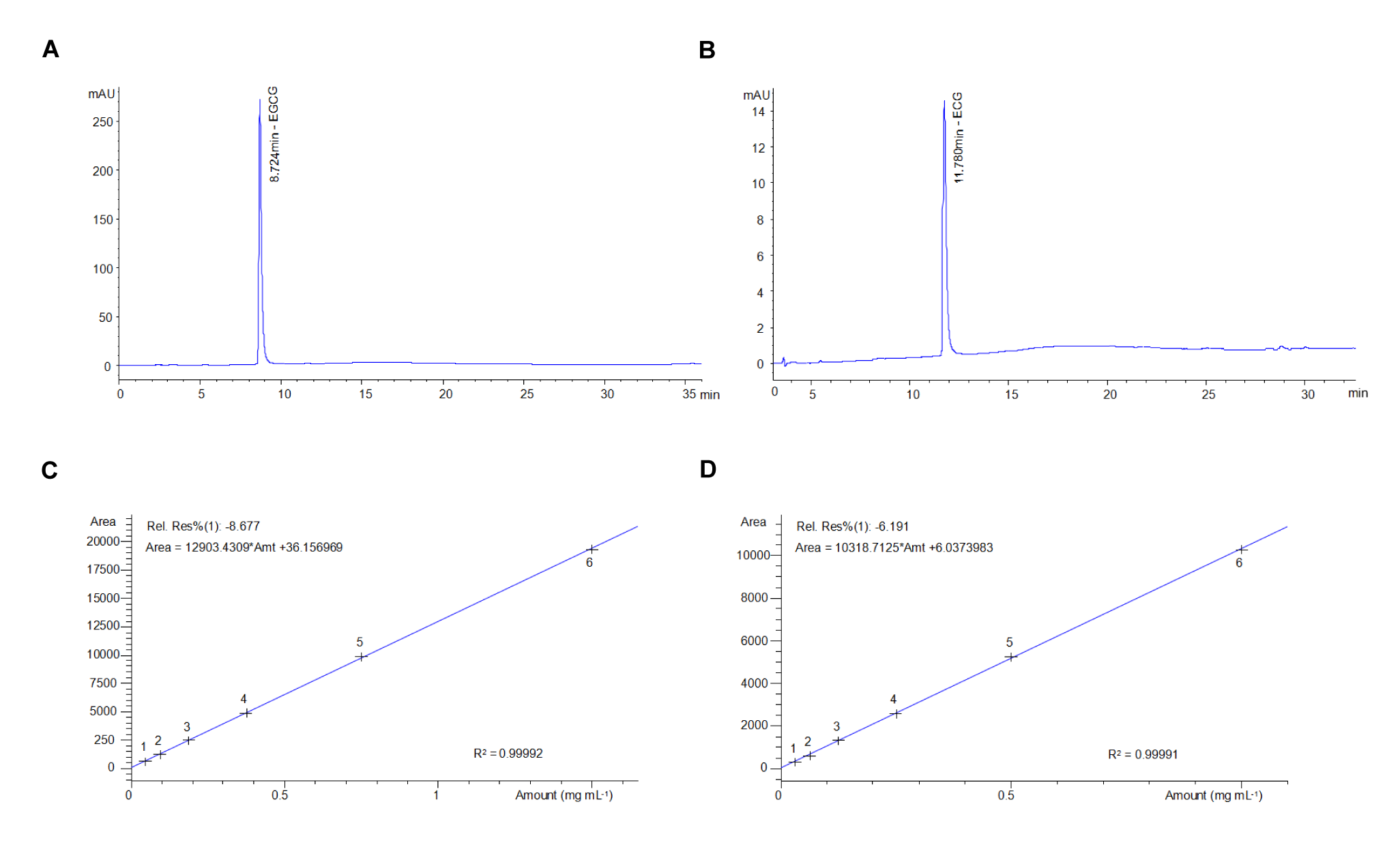

Supplement: Supplementary file 1 [file Presentation_1.ZIP › Supplementary Material/Supplementary figure 2.tif]

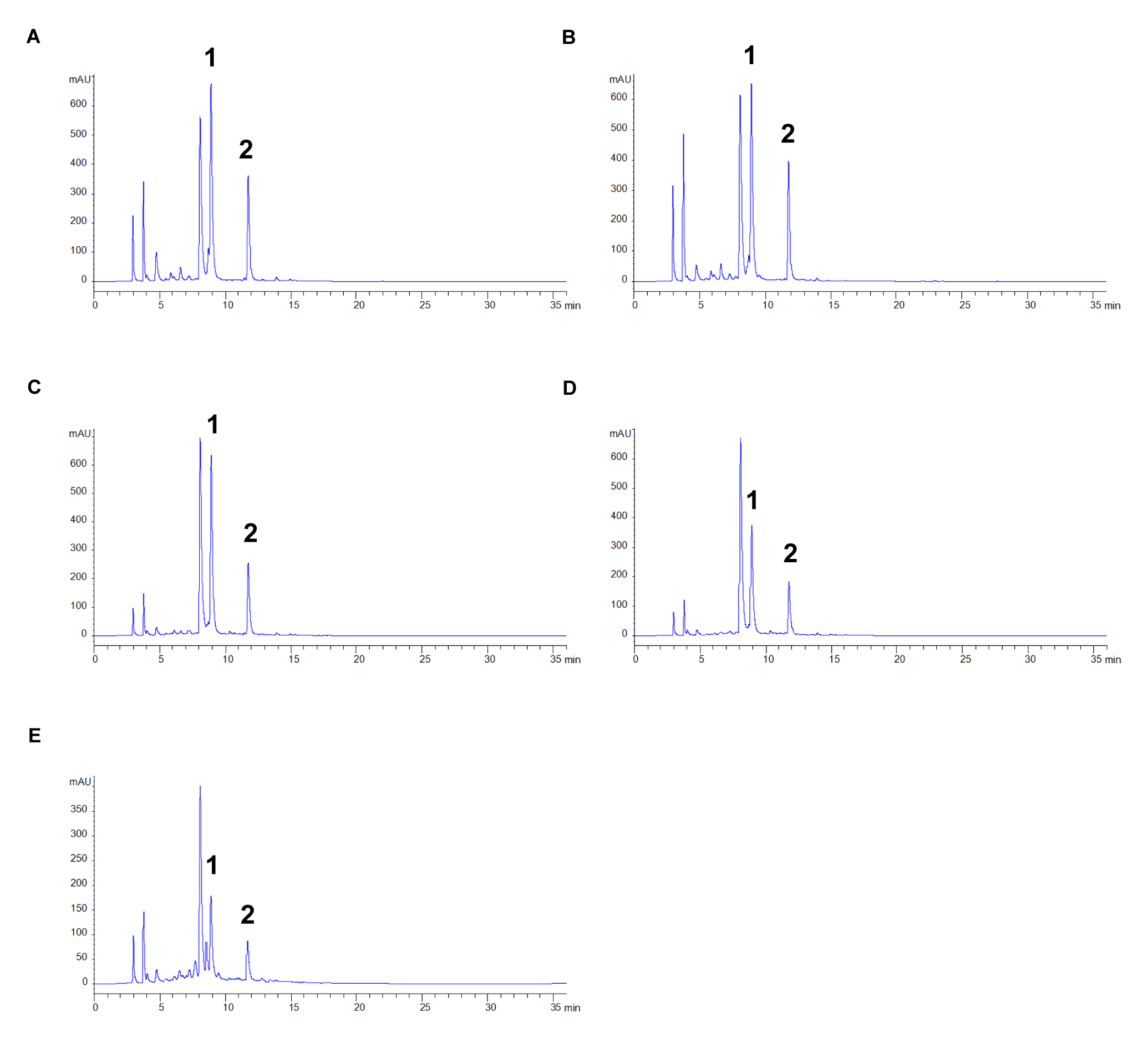

Supplement: Supplementary file 1 [file Presentation_1.ZIP › Supplementary Material/Supplementary figure 3.tif]
