# Supplementary material for: Subcellular Localization of Galloylated Catechins in Tea Plants [Camellia sinensis (L.) O. Kuntze] Assessed via Immunohistochemistry
Source: Front Plant Sci. 2016 May 26;7:728. doi: 10.3389/fpls.2016.00728 (PMC4881381; doi:10.3389/fpls.2016.00728)
Supplement: Supplementary file 1 [file Presentation_1.ZIP › Supplementary Material/Supplementary Material.doc]

Supplementary Material

**Subcellular Localization of Galloylated Catechins in Tea Plants (*Camellia sinensis* (L.) O. Kuntze) Assessed via Immunohistochemistry**

**Huanhuan Xu^1,2,3¶^, Ya Wang^1,2,3¶^, Yana Chen^1,2^, Pan Zhang^1,2^, Yi Zhao^1,2^, Yewei Huang^1,2,3*^, Xuanjun Wang^1,2,4,5*^, Jun Sheng^1,2,5*^**

*** Correspondence:** Corresponding Author: [shengj@ynau.edu.cn](mailto:shengj@ynau.edu.cn); [wangxuanjun@gmail.com](mailto:wangxuanjun@gmail.com); [lichuangyewei100@163.com](mailto:lichuangyewei100@163.com).

^¶^These authors contributed equally to this work.

**Supplementary Figure 1. Amino acid sequence of the kappa chain variable region of the P6C2 antibody.**

**Supplementary Figure 2. HPLC graphs and calibration curves of standard galloylated catechins. (A)** HPLC graph of EGCG reference standards. Retention time: 8.724min; **(B)** HPLC graph of ECG reference standards. Retention time: 11.780min. **(C)** Calibration curve of EGCG. The EGCG concentrations are: 1.5, 0.75, 0.375, 0.1875, 0.09375, 0.046875 mg mL-1. Equation: Area = 12903.4309*Amt +36.156969. R2 = 0.99992; **(D)** Calibration curve of ECG. The ECG concentrations are: 1, 0.5, 0.25, 0.125, 0.0625, 0.03125 mg mL-1. Equation: Area = 10318.7125*Amt +6.0373983. R2 = 0.99991.

**Supplementary Figure 3. Chromatograms of HPLC analysis of galloylated catechins from different tea tissues. (A)** first leaf; **(B)** bud; **(C)** second leaf; **(D)** third leaf; **(E)** tender stem. Peaks: 1, EGCG; 2, ECG.
